# Case report: Pallidal deep brain stimulation for treatment of tardive dystonia/dyskinesia secondary to chronic metoclopramide medication

**DOI:** 10.3389/fneur.2022.1076713

**Published:** 2023-01-12

**Authors:** Johanna M. Nagel, Joseph Ghika, Joachim Runge, Marc E. Wolf, Joachim K. Krauss

**Affiliations:** ^1^Department of Neurosurgery, Hannover Medical School, Hannover, Germany; ^2^Service de Neurologie, Hôpital du Valais, Sion, Switzerland; ^3^Department of Neurology, Neurozentrum, Klinikum Stuttgart, Stuttgart, Germany; ^4^Department of Neurology, Universitätsmedizin Mannheim, University of Heidelberg, Mannheim, Germany

**Keywords:** pallidal DBS, metoclopramide, tardive dystonia, tardive dyskinesia, GPi DBS, case report

## Abstract

**Objectives:**

Tardive dystonia/dyskinesia (TDD) occurs as a side effect of anti-dopaminergic drugs, including metoclopramide, and is often refractory to medication. While pallidal deep brain stimulation (DBS) has become an accepted treatment for TDD secondary to neuroleptic medication, there is much less knowledge about its effects on metoclopramide-induced TDD.

**Methods:**

We present the case of a woman with metoclopramide-induced TDD, whose symptoms were initially misjudged as “functional.” After 8 years of ineffective medical treatments, she received bilateral implantation of quadripolar electrodes into the posteroventral lateral globus pallidus internus (GPi).

**Results:**

GPi DBS led to significant symptom reduction [Burke–Fahn–Marsden Dystonia Rating Scale (BFMDRS) motor score 24/44 at admission and 7/44 at discharge]. Chronic stimulation led to full recovery from TDD symptoms 9 years after surgery. The BFMDRS motor score decreased to 0.5 (98% improvement).

**Discussion:**

Pallidal DBS may result in sustained improvement of TDD secondary to chronic metoclopramide intake in the long term.

## 1. Introduction

Deep brain stimulation (DBS) of the globus pallidus internus (GPi) has become the standard treatment for dystonia (1, 2). Several studies have shown that DBS is similarly effective in patients with tardive dystonia/dyskinesia (TDD) as compared to inherited or idiopathic dystonia (3–7). While TDD has been a common adverse effect of neuroleptic treatment, it may rarely be secondary to chronic medication with metoclopramide (8, 9). Thus far, no detailed information has become available on the effect of GPi DBS for the treatment of metoclopramide-induced TDD. Only data from three patients have been reported so far, but individual outcomes were not mentioned in the case series. We therefore, would like to present a case of metoclopramide-induced TDD with dystonia and dyskinesia after metoclopramide administration.

## 2. Case report

A 40-year-old woman presented with a 7-year history of TDD after chronic medication with metoclopramide. She had a history of migraine, gluten intolerance, and Crohn's disease. At 32 years of age, she was treated with metoclopramide for nausea and gastroparesis. About a year on metoclopramide medication, she developed oculogyric crises, and dystonic/dyskinetic involuntary movements of orofacial muscles, tongue, trunk, and scalp. Several months later, retrocollis and dystonic movements of the right leg appeared. She had never received metoclopramide or any other dopamine-blocking medication before. The hyperkinetic movements had started after months of chronic metoclopramide intake and before intake of any neuroleptics. The patient had no family history of dystonia and/or dyskinesias and moreover, no family history of any movement disorder, including Parkinson's disease or tremor. The diagnosis of metoclopramide-induced TDD was made after a neurological workup, including MRI, lumbar puncture, blood tests, and jejunal biopsy, all of which showed negative results. After receiving the diagnosis of metoclopramide-induced TDD, metoclopramide was withdrawn. Then, she received multiple medications for the treatment of TDD, including neuroleptics (dipiperon, risperidone, clozapine, and olanzapine), for several months without a clear effect on the movement disorder. Although the dyskinesias worsened over time, the patient did not feel a worsening or improving effect linked to the neuroleptic medication. After a trial with tetrabenazine, dyskinesias were slightly worse transiently. Benzodiazepines (oxazepam, clonazepam, and clobazam), antidepressants (mirtazapine, fluoxetine, and trimipramine), and other medications (tizanidine, baclofen, dantrolene, and buspirone) were administered without any improvement. The patient had botulinum toxin injections to the facial muscles and scalp several times along the course of the disease. She reported no improvement thereafter. At some point, the symptoms were classified as “functional” and she was treated in different psychiatric hospitals for depression and fatigue without any improvement.

At 40 years of age, she presented at the author's institution for the first time. At that time, she was unable to work or do housework, lived with her mother, and had suffered from insomnia and depression. The neurological examination revealed marked phasic dystonic/dyskinetic movements of facial muscles including blepharospasm, orofacial dyskinesias with tongue movements, involvement of occipital and cervical muscles with prominent rippling scalp movements, retroflexion and turn of the head, dystonic movements of her upper trunk, and posturing of the right foot resulting in abnormal gait. No deficits of the cranial nerves or sensorimotor dysfunction were detected. The motor score on the Burke–Fahn–Marsden Dystonia Rating Scale (BFMDRS) was 24. The patient was psychiatrically stable, especially without suicidal thought. The Hamilton Depression Score (HAMD) did not indicate subclinical or overt depression.

**Figure 1:**
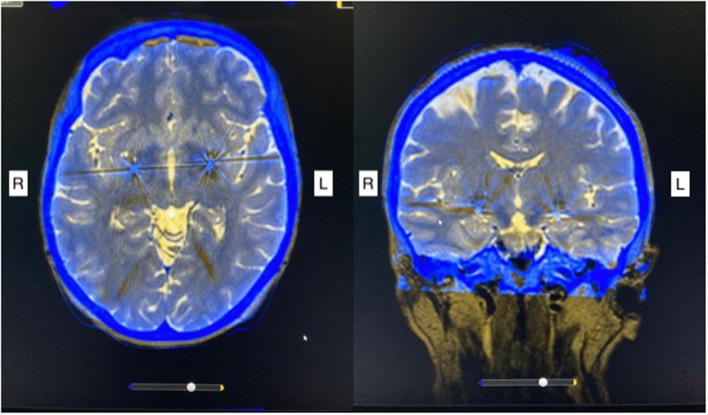
Post-operative stereotactic CT imaging fused with preoperative MRI scan shows bilateral electrode placement in the posteroventral lateral globus pallidus internus. R, right; L, left.

She underwent the implantation of quadripolar DBS electrodes (Medtronic 3387, four contacts with a length of 1.5 mm per contact and spacing of 1.5 mm between contacts) bilaterally in the posteroventral lateral GPi guided by CT-stereotactic surgery and microelectrode recording under local anesthesia with techniques, as described in detail elsewhere (4, 10). After postoperative stereotactic CT-imaging confirmed the appropriate placement of the DBS electrodes (Figure 1), she received a non-rechargeable implantable pulse generator (IPG) (Medtronic, Activa PC). Automatic lead detection in postoperative CT scans showed the following coordinates for the lowest contact: Right GPi: x = 20.4, y = 4.1, z = −4.8; Left GPi: x = 19.6, y = 2.5, z = −3.9. During early programming, dystonia of the leg disappeared completely and the other involuntary movements were markedly improved, but the patient still felt considerable tension in the neck and scalp area. The stimulation settings upon discharge are shown in Table 1. The motor BFMDRS was reduced to seven (71% improvement).

**Table 1:** Stimulation settings at discharge, 20 months post-surgery, and 9 years post-surgery.

**Time point**	**Stimulation site**	**Active contacts**	**Amplitude**	**Pulse width**	**Frequency**
At discharge	R GPi	1 (–), 2 (+)	4.8 V	210 μs	130 Hz
	L GPi	10 (+)	2.5 V	240 μs	130 Hz
20 months post-surgery	R GPi	0 (–), 1 (–), 2 (–)	1.1 V	450 μs	125 Hz
	Interleaving	3 (–)	2.3 V	450 μs	125 Hz
	L GPi	10 (–), 11 (–)	1.8 V	450 μs	125 Hz
	Interleaving	8 (–), 9 (–)	1.7 V	450 μs	125 Hz
9 years post-surgery	R GPi	0 (–), 1 (–), 2 (–)	1.2 V	450 μs	125 Hz
	Interleaving	3 (–)	4.3 V	450 μs	125 Hz
	L GPi	10 (–), 11 (–)	3.3 V	450 μs	125 Hz
	Interleaving	8 (–), 9 (–)	1.8 V	450 μs	125 Hz

At 20 months post-surgery, the stimulation was switched to an interleaving program. Contacts were named 0–3 on the right hemisphere and 8–11 on the left hemisphere with contacts 0 and 8 as the most ventral contacts.

GPi, globus pallidus internus.

Within the next few months, several stimulation settings were tested and the dystonic/dyskinetic movements slowly improved further. The motor BFMDRS at the 1-year follow-up was 1 (96% improvement). Two years postoperatively, the IPG was replaced by a rechargeable device. With continued adjustment of stimulation settings (refer to Table 1) and chronic stimulation, the involuntary movements almost completely subsided. At 9-year follow-up, at 49 years of age, the patient was living with a partner, worked full time, and took care of her family. She continued with chronic DBS with relatively high energy delivery without side effects (refer to Table 1). The motor BFMDRS was 0.5 (98% improvement).

## 3. Discussion

While the frequency of TDD, in general, has declined over the years with the use of second-generation neuroleptics, they still may present a debilitating side effect (3). If symptoms are recognized early and treated appropriately, improvement ranging between 62 and 76% can be achieved (7). Although metoclopramide was found to be the second most common drug after haloperidol to cause TDD (8), according to newer data, the risk for the occurrence of TDD after metoclopramide intake might have been overrated. A recent review found the risk of TDD under metoclopramide medication to be around 0.1% per 1,000 patient-years (9). Nevertheless, the FDA recommends that the chronic use of metoclopramide medication should be avoided.

Tardive dystonia/dyskinesia has been reported to respond well to pallidal DBS yielding a 76% improvement of the BFM at a mean follow-up of 25.6 months according to a recent meta-analysis (7). In a multicenter randomized controlled trial, dystonia severity had improved significantly by 23% at 3 months of chronic stimulation and by 42% at 6 months with infrequent and transient side effects (6). However, it has been problematic that the degree of response to pallidal DBS varies widely in patients with TDD (11), possibly due to causative drug. Those studies including patients with metoclopramide-induced TDD showed better results on short-term (56% improvement in BFMDRS at 3 months and 40% on the ESRS) and long-term follow-up, ranging between 60 and 83% (5, 6). However, our patient improved even better in short term (71% improvement in BFMDRS at discharge and 96% improvement at 1-year follow-up as compared to baseline).

Our case also demonstrates that the beneficial effects of pallidal DBS in metoclopramide-induced TDD can be sustained in the long term. Thus far, only summarized data of a total of three patients with metoclopramide-induced TDD have been reported in two previous series on pallidal DBS for TDD. The mean improvement of TDD in a series of 19 patients including two patients with metoclopramide-induced TDD reported by Pouclet-Courtemanche et al. (5) at 12 months of chronic DBS was 58% on the Extrapyramidal Symptoms Rating Scale (ESRS). The third patient, reported in the series of Gruber et al. (3), also benefitted from stimulation and experienced a rapid worsening of symptoms by 80% when stimulation was turned off. Again, no detailed information is available on the clinical improvement in this patient, but the overall improvement in this series was 59% on the BFMDRS at the last follow-up (3). Notably, all patients who underwent pallidal DBS for metoclopramide-induced TDD were female. They had received metoclopramide for the duration of several months up to 4 years before the development of TDD (5, 6).

It is noteworthy that the patient presented with right foot involvement, as tardive dyskinesias usually manifest in the face, head, and upper extremities (12, 13). Lower extremity involvement is more typical in genetic dystonias (14). Since the patient did not undergo genetic testing and also Levodopa was not tried to exclude L-Dopa-responsive dystonia, an additional underlying genetic cause, even if rare, cannot be completely excluded. However, the clinical presentation of the patient does not coincide with the phenotypes of the more common genotypes of dystonia. A limitation of this and also of the previous case reports that underwent DBS is the lack of information on the exact dosages and duration of medications, including the dose of metoclopramide. Other authors, however, had communicated that the risk for the development of tardive dystonia/dyskinesia is given, when the oral dose exceeds 10 mg 3–4 times daily (9, 15, 16).

Another remarkable finding in our patient, besides the peculiar dyskinesias of the scalp muscles, was the early response to pallidal DBS concerning mainly the phasic elements of dystonia. We demonstrated a major short- and long-term improvement of metoclopramide-induced TDD under pallidal DBS. However, experience with the effects of pallidal DBS in metoclopramide-induced TDD is needed. The limited data which are available, however, may suggest that these patients respond particularly well to chronic stimulation.

## Data availability statement

The original contributions presented in the study are included in the article, further inquiries can be directed to the corresponding author.

## Ethics statement

Ethical review and approval was not required for this case report in accordance with the local legislation and institution requirements. The patient provided written informed consent for the use of anonymized data for research purposes and for publication of any potentially identifiable images or data included in this article.

## Author contributions

JN: data and material collection in research project and writing, review, and editing of the manuscript. JG: concept and data and material collection in research project and writing, review, and editing of the manuscript. JR and MW: data and material collection in research project and review and editing of the manuscript. JK: concept, supervision, data and material collection in research project, and review and editing of the manuscript. All authors contributed to the article and approved the submitted version.
